# Children's eating behaviour: A comparison between normal, overweight and obese children

**DOI:** 10.1016/j.amsu.2022.104890

**Published:** 2022-11-13

**Authors:** Lily Shuzeen Kimin, Constance Liew Sat Lin, Richard Avoi, Firdaus Hayati, Mohd Nazri Mohd Daud, Symeon Mandrinos, Alvin Oliver Payus

**Affiliations:** a Hospital Kuala Penyu, WDT No 35, 89740, Kuala Penyu, Sabah, Malaysia; b Faculty of Medicine and Health Sciences, Universiti Malaysia Sabah (UMS), Jalan UMS, 88400, Kota Kinabalu, Sabah, Malaysia; c Swinburne University of Technology, Q5B, 93350, Kuching, Sarawak, Malaysia

**Keywords:** Pediatric obesity, Overweight, BMI, Nutritional status, Feeding behaviour

## Abstract

**Background:**

Childhood obesity has become a major global health concern and has been increasing dramatically over the years. Previous study has shown that specific eating behaviours may have been associated with obesity especially under-responsiveness to internal satiety cues and over-responsiveness to external food cues such as the taste, smell, availability and emotions. However, there is still inadequate number of studies present to describe the association between the children's body mass index (BMI) and their eating behaviours, especially in Sabah, Malaysia. Therefore, the objective of this study is to established the association between the children's eating behaviours with their nutritional status based on their body mass index.

**Materials and methods:**

A cross-sectional study involving 484 children aged 6–12 years old was conducted in Kota Kinabalu, which is a developing urban area in Sabah. The children were recruited from five primary schools that were selected based on multistage stratified and convenience sampling method. Sociodemographic details and anthropometric measures both parents and children, and eating behaviours of children were assessed using Children Eating Behaviour Questionnaires (CEBQ). Age-adjusted BMI z-scores were then calculated according to the World Health Organization recommendations to assess nutritional status.

**Results:**

The prevalence of childhood obesity among children aged 6–12 years old is 13.2%. The mean scores of ‘Food Approach’ subscales from the CEBQ showed higher mean score in overweight and obese groups as compared to the mean score in normal weight group. The mean scores of ‘Food Avoidance’ subscales showed lower mean score in overweight and obese groups as compared to mean score in normal weight group.

**Conclusion:**

This study provides evidence that childhood obesity is yet to be a prevalent health problem in a developing urban area considering the “Food approach” subscales were positively associated with the excess weight in children.

## Highlights

• Children's eating behaviours with their nutritional status based on their body mass index.
• Childhood obesity is yet to be a prevalent health problem.
• “Food approach” subscales were positively associated with the excess weight in children.

## 1 Introduction

Obesity is a chronic disorder which have significant impact on both physical and psychological health. Childhood obesity has been rising dramatically over the years and has become a major concern. The latest statistic from the National Health and Morbidity Survey (NHMS) in Malaysia shows that the national prevalence of obesity in 2019 was 14.8%, which has increased from 11.9% in 2015 [1,2]. This is worrying because overweight and obesity are considered risk factors for various chronic diseases such as type 2 diabetes, high blood pressure, heart diseases, stroke, and some forms of cancer. Furthermore, overweight or obese child are likely to be overweight or obese in their adulthood. Therefore, are at greater risk of developing chronic cardiovascular diseases [3].

While it is agreed that the energy intake exceeds the energy expenditure in overweight children, less is known about the specific behaviours involved. Studies have shown differences in several dimensions of eating behaviours among children [4]. According to Roy S et al. (2011) [5], specific eating behaviours that have been associated with obesity include under-responsiveness to internal satiety cues (low satiety responsiveness, high speed of eating) and over-responsiveness to external food cues such as taste, smell, availability and emotions (high enjoyment of food, food responsiveness and emotional overeating). On the other hand, underweight children seem to be more selective in relation to food, consuming small meals, with a limited number of foods and more slowly, thus reflecting a lack of interest in food [4].

It is known that eating behaviors are developed in the first years of life and will influence the habits in adulthood [6]. Eating behaviours established in childhood persist, with implications such as fussiness and poor dietary variety, or high responsiveness to food cues and increased obesity risk [7]. The study of children's behaviour should thus be seen as a starting point for targeted and effective nutrition education programs, while at the same time suggesting further research strategies to elucidate the interactions between the various factors influencing children's eating behaviours [7]. Yet there is inadequate number of studies in this part of the world to describe the association of BMI with the eating behaviours of primary school children. Thus, the aim of the present study is to determine the prevalence of obesity and investigate the association between child eating behaviors and body weight status in a developing urban are in Malaysia.

## 2 Methodology

### 2.1 Populations and sample sizes

The participants of the study were children aged 6–12 years old and their parent provided that their parent consented to be include and the children did not fulfill any exclusion criteria. The exclusion criteria were; i) Children with chronic diseases that will affect their metabolism, appetite and body weight such as type I diabetes mellitus, childhood asthma, nephrotic syndrome, and others. ii) Children who is on long-standing medication that will affect the body weight such as corticosteroids and others.

The primary schools provide an excellent setting for the recruitment of children in the targeted age group because the compulsory schooling age is six years old according to the law in Malaysia. Five national primary schools were selected within the city of Kota Kinabalu based on multistage stratified sampling method. One school from each zone was selected by simple random sampling method using cards. Every school has an equal chance and likelihood of being selected in the sample. In each school, participants from standard one (age six and seven years old) to standard six (age 12 years old) were selected using convenience sampling. Convenience sampling was used in view of each school has different number of students attending and difficulty to get full cooperation from respondent to fill up the questionnaire. The sample size was calculated by taking the confidence level at 95%, anticipated frequency of 80%, and the prevalence rate of overweight of 0.20 based on a study by Loh DA et al. (2013) [8] among secondary school students in the state of Selangor, Malaysia. The minimum sample size required was 426, but a non-response rate of 10% were added, making the final sample size 468 subjects.

### 2.2 Study instruments

This research involves two type of data collections, which were questionnaire and anthropometry measurement. The first part of the questionnaire involves mainly the sociodemographic data which include the children's age, gender, weight, height, and ethnicity. The parent's age, highest educational level, current occupation and monthly total family income were also taken. The second part focused on the children's eating behaviours which were adapted from the Children eating behaviour questionnaire (CEBQ) which was translated and validated in Bahasa Malaysia [8] and English [9]. It consists of 35 item questions using Likert scale, with the score ranging from 1 to 5: never (1), rarely (2), sometimes (3), often (4) and always (5). It assessed outcomes 8 subscales eating behaviours which belong to food avoidance [Food Responsiveness (FR), Enjoyment of Food (EF), Emotional Overeating (EOE), Desire to Drink (DD)] and food avoidance [Satiety Responsiveness (SR), Slowness in Eating (SE), Emotional Undereating (EUE) and Food Fussiness (FF)]. It was assessed through the subjective perception of parents about their children's behaviour. The scores of questions from the same subscale were added up, so that each subscale had a mean value and standard deviation.

The anthropometry measurement involves the children and their parent's height and weight which were recorded by trained personnel. The body mass index (BMI) was measured based on the calculation of each participant's height and weight using the standard formula (weight in kg)/height in meter 2). The BMI of the children were categorized using the World Health Organization BMI-for-age z-scores graft chart (5–19 years old). Weight of the parents were deduced from formula of BMI.

### 2.3 Statistical analysis

The data were analyzed using a statistical software platform (IBM® SPSS ver 25.0). Descriptive measures like mean, standard deviation, and percentage were used to describe the socio-demographic, parent income, educational level, occupation and distribution of each CEBQ subscales. Physical measures were described by calculating the descriptive measures like mean and standard deviation for height, weight, and BMI percentile for the children and their parent.

## 3 Results

### 3.1 Demographic data

There were 484 subjects included in this study where 198 (40.9%) were male and 286 (59.1%) were female participants. The mean age for the male group was 9.73 ± 2.42, while the female group was 8.05 ± 1.88. There was a significant different of mean body mass index (BMI) for male group (24.3 ± 4.05) as compare to the female group (17.9 ± 3.62). The characteristics of the child participants which include gender, age, weight, height and BMI are summarized in Table 1.

**Table 1:** Mean distribution of child participants (gender, mean age and BMI).

Variables	Male	Female
Gender [n (%)]	198 (40.9%)	286 (59.1%)
AGE [Mean (SD)]	9.73 (SD 2.42)	8.05 (SD 1.88)
WEIGHT [kg (SD)]	35.65 (SD 11.49)	29.85 (SD 10.29)
HEIGHT [meter (SD)]	136.16 (SD 50.92)	123.65(SD 48.93)
BMI (SD)	24.3 (SD 4.05)	17.9 (SD 3.62)

Table 1 showed the characteristics of the 484 children included in the study which include gender, age, weight, height and BMI. There were 198 (40.9%) male participants with the mean age of 9.73 ± 2.42, and 286 (59.1%) female participants with the mean age of 8.05 ± 1.88. There was a significant different of mean BMI for male group (24.3 ± 4.05) as compare to the female group (17.9 ± 3.62). BMI: Body Mass Index; SD: Standard Deviation.

There was a significant different in the education level among the parents of the children in this study. Among the father, 239 (50.5%) were secondary graduates and 234 (49.4%) received tertiary education. As for the mother, 13 (2.7%) received primary education, 229 (47.3%) received secondary education and 242 (50%) received tertiary education. There were higher proportion of self-employed among the mothers and non-government occupations among fathers of participants. There were 135 (28.5%) government occupations, 268 (56.7%) non-governmental occupations and 70 (14.8%) self-employed occupations among fathers versus 160 (33.1%) government occupations, 146 (30.2%) non-governmental occupations and 178 (36.8%) self-employed occupations among mothers. The summary of the parent's BMI and sociodemographic distribution focusing on their education level and occupation are presented in Table 2.

**Table 2:** BMI and sociodemographic distribution of the parent (education level and occupation).

Variables	Frequency (N)	Percentage (%)
Parent BMI		
Mother		
Underweight	5	1.0
Normal	216	44.6
Overweight	215	44.4
Obese	48	9.9
Father		
Underweight	1	0.2
Normal	114	24.1
Overweight	315	66.6
Obese	43	9.1
Parent education		
Primary education		
Mother	13	2.7
Father	–	–
Secondary education		
Mother	229	47.3
Father	239	50.5
Tertiary education		
Mother	242	50.0
Father	234	49.4
Parent occupation		
Government		
Mother	160	33.1
Father	135	28.5
Non- Government		
Mother	146	30.2
Father	268	56.7
Self-employed		
Mother	178	36.8
Father	70	14.8

Table 2 showed the BMI and socio demographic distribution of parent focusing on the education level and occupation. Out of the 484 mothers, 216 (37.6%) were in a normal range BMI and 215 (37.5%) were overweight. Among fathers, higher number of overweight 315 (54.9%) followed by normal range of BMI 114 (19.5%). BMI: Body Mass Index.

### 3.2 Prevalence of obesity

In this study, the BMI of 64 (13.2%) out of 484 children were in the category of obesity (Z-score = 2) (95% CI = 11.45–15.35; range BMI = 15.1–24.9). Among the 64 children, 36 (56.3%) were male and 28 (43.7%) were female. 65 (13.4%) of the participants were found to be in the category of overweight (Z-score = 1) (95% CI = 11.36–16.79; range BMI = 20.1–35.1), 334 (69%) in the category of normal BMI (Z-score = 0), 16 (3.3%) participants in the category of thinness (Z-score = −2) and 5 (1.1%) were in the category of severe thinness (Z-score = −3).

### 3.3 Mean scores and distributions of each CEBQ subscales

Each score of the Children eating behaviour questionnaire (CEBQ) subscales was further divided into three categories which were low (scale 1–2), medium (scale 3–4) and high (scale 5). The range of mean score for each subscale was 0.55 (Min 2.61; Max 3.16). The distribution of scores for the ‘Food Approach’ subscales (FR, EF, EOE, DD) showed the highest among the low category, followed by medium and high category. As for the ‘Food Avoidance’ subscales (SR, EUE, FF), the highest distribution was the medium category, followed by low category and high category. Table 3 summarizes the mean scores and distributions of each CEBQ subscales.

**Table 3:** Mean scores and Distributions of CEBQ subscales.

CEBQ Subscales	N (Low, Medium, High)	Mean	Standard Deviation	% (Low, Medium, High)
‘Food Approach’ subscales				
**FR**	484	2.91	0.99	L: 56.4
	(273,99,112)			M: 20.5
				H: 23.1
**EF**	484	3.16	0.95	L: 46.5
	(225,131,128)			M: 27.1
				H: 26.4
**EOE**	484	2.61	0.80	L: 66.3
	(321,122,41)			M: 25.2
				H: 8.5
**DD**	484	2.86	0.95	L: 58.9
	(285, 94, 105)			M: 19.1
				H: 21.7
**‘Food Avoidance’ subscales**				
**SR**	484	2.95	0.61	L: 47.7
	(231,231, 22)			M: 47.8
				H: 4.5
**SE**	484	2.69	0.81	L: 57.6
	(279, 163, 42)			M: 33.7
				H: 8.7
**EUE**	484	2.94	0.46	L: 41.9
	(203,261,20)			M: 54
				H: 4.1
**FF**	484	3.02	0.45	L: 40.1
	(194,274,16)			M: 56.6
				H: 3.3

Table 3 showed the mean scores and distributions of CEBQ subscales. Each score of the CEBQ subscales was further divided into three categories which were low (scale 1–2), medium (scale 3–4) and high (scale 5). The range of mean score for each subscale was 0.55 (Min 2.61; Max 3.16). The distribution of scores for the ‘Food Approach’ subscales (FR, EF, EOE, DD) showed the highest among the low category, followed by medium and high category. As for the ‘Food Avoidance’ subscales (SR, EUE, FF), the highest distribution was the medium category, followed by low category and high category. CEBQ: Children Eating Behaviour Questionnaires; DD: Desire to Drink; EF: Enjoyment of Food; EOE: Emotional Overeating; EUE: Emotional Undereating; FF: Food Fussiness; FR: Food Responsiveness; SE: Slowness in Eating; SR: Satiety Responsiveness.

The mean scores of the ‘Food approach’ subscale in age group of 6–7 years old were lower compared to the other three groups. As for the ‘Food Avoidance’ subscales, the mean scores of all age groups were within 2.06–2.75. Children at the age group of 12 years old has the lowest scores among the other three age groups. The mean scores of four categories of age groups in CEBQ subscales were shown in Table 4.

**Table 4:** Mean Scores of 4 categories of age groups in CEBQ Subscales.

CEBQ Subscales	Age Group	N	Mean Score	SD
**‘Food Approach’ subscales**				
**FR**	**Age 6**–**7**	87	2.01	0.94
	**Age 8**–**9**	126	2.29	0.92
	**Age 10**–**11**	140	2.66	1.07
	**Age 12**	131	2.95	0.80
**EF**	**Age 6**–**7**	87	2.38	0.87
	**Age 8**–**9**	126	2.55	0.86
	**Age 10**–**11**	140	2.89	0.95
	**Age 12**	131	3.05	0.75
**EOE**	**Age 6**–**7**	87	1.83	0.75
	**Age 8**–**9**	126	2.04	0.80
	**Age 10**–**11**	140	2.35	0.91
	**Age 12**	131	2.53	0.78
**DD**	**Age 6**–**7**	87	2.07	0.93
	**Age 8**–**9**	126	2.29	0.85
	**Age 10**–**11**	140	2.64	1.06
	**Age 12**	131	2.85	0.81
**‘Food Avoidance’ subscales**				
**SR**	**Age 6**–**7**	87	2.60	0.69
	**Age 8**–**9**	126	2.54	0.65
	**Age 10**–**11**	140	2.41	0.73
	**Age 12**	131	2.58	0.51
**SE**	**Age 6**–**7**	87	2.06	0.79
	**Age 8**–**9**	126	2.29	0.85
	**Age 10**–**11**	140	2.39	0.86
	**Age 12**	131	2.56	0.80
**EUE**	**Age 6**–**7**	87	2.75	0.55
	**Age 8**–**9**	126	2.71	0.55
	**Age 10**–**11**	140	2.59	0.59
	**Age 12**	131	2.48	0.53
**FF**	**Age 6**–**7**	87	2.67	0.56
	**Age 8**–**9**	126	2.67	0.55
	**Age 10**–**11**	140	2.60	0.62
	**Age 12**	131	2.57	0.52

Table 4 showed the summary of the mean scores of the four categories of age groups in each CEBQ subscales. The mean scores of the ‘Food approach’ subscale (FR, EF, EOE, DD) in age group of 6–7 years old were lower compared to the other three groups, while for the ‘Food Avoidance’ subscales (SR, SE, EUE, FF), children at the age group of 12 years old has the lowest scores among the other three age groups. CEBQ: Children Eating Behaviour Questionnaires; DD: Desire to Drink; EF: Enjoyment of Food; EOE: Emotional Overeating; EUE: Emotional Undereating; FF: Food Fussiness; FR: Food Responsiveness; SD: Standard Deviation; SE: Slowness in Eating; SR: Satiety Responsiveness.

The mean scores of ‘Food Approach’ subscales (FR, EF, EOE, DD) showed higher mean score in overweight and obese groups as compared to mean score in normal weight group. The mean scores of ‘Food Avoidance’ subscales (SR, EUE, FF) showed lower mean score in overweight and obese groups as compared to mean score in normal weight group. The SE subscale from the ‘Food Avoidance’ showed the mean scores of overweight and obese groups were higher as compared to normal weight group. The summary of the mean scores of the three groups of the nutritional statuses of the children (normal weight, overweight and obese) in each CEBQ subscales are shown in Table 5.

**Table 5:** Mean Scores of three groups of children BMI in CEBQ Subscales.

Nutritional Status	N	Mean	SD
‘Food Approach’ subscales			
FR			
**Nor**	334	2.47	0.56
**Owt**	65	4.14	0.34
**Obe**	64	4.44	0.39
**EF**			
**Nor**	334 65 64	2.72 4.47 4.55	0.52 0.29 0.33
**Owt**			
**Obe**			
**EOE**			
**Nor**	334	2.29 3.50 3.76	0.52 0.43 0.38
**Owt**	65		
**Obe**	64		
**DD**			
**Nor**	334	2.44	0.50
**Owt**	65	4.05	0.34
**Obe**	64	4.32	0.41
**‘Food Avoidance’ subscales**			
**SR**			
**Nor**	334	3.00	0.44
**Owt**	65	2.80	0.43
**Obe**	64	2.35	0.69
**SE**			
**Nor**	334	2.38	0.63
**Owt**	65	3.31	0.47
**Obe**	64	3.79	0.63
**EUE**			
**Nor**	334	3.04	0.36
**Owt**	65	2.53	0.28
**Obe**	64	2.55	0.31
**FF**			
**Nor**	334	3.07	0.37
**Owt**	65	2.67	0.36
**Obe**	64	2.81	0.49

Table 5 showed the summary of mean scores of the three groups of the nutritional statuses of the children (normal weight, overweight and obese) in each CEBQ subscales. The mean scores of ‘Food Approach’ subscales (FR, EF, EOE, DD) showed higher mean score in overweight and obese groups as compared to mean score in normal weight group. The mean scores of ‘Food Avoidance’ subscales (SR, EUE, FF) showed lower mean score in overweight and obese groups as compared to mean score in normal weight group. The SE subscale from the ‘Food Avoidance’ showed the mean scores of overweight and obese groups were higher as compared to normal weight group. BMI: Body Mass Index; CEBQ: Children Eating Behaviour Questionnaires; DD: Desire to Drink; EF: Enjoyment of Food; EOE: Emotional Overeating; EUE: Emotional Undereating; FF: Food Fussiness; FR: Food Responsiveness; Nor: Normal; Obe: Obese; Owt: Overweight; SD: Standard Deviation; SE: Slowness in Eating; SR: Satiety Responsiveness.

### 3.4 Association between CEBQ subscales and children BMI

We divided the nutritional status into three groups based on their body mass index (BMI), which are normal, overweight and obese. Thereafter, we compare the mean scores of the CEBQ subscales between the nutritional status groups and also within the group itself. Both the ‘Food Approach’ and ‘Food Avoidance’ subscales were significant in association with three nutritional statuses of participants. The association between the CEBQ subscales and the BMI are summarized in Table 6.

**Table 6:** Association between CEBQ subscales and children BMI.

Nutritional Status	Mean	SD	F	Sig
‘Food Approach’ subscales				
FR				
**Nor**	2.47	0.56	579.56	0.00
**Owt**	4.14	0.34		
**Obe**	4.44	0.39		
**ER**				
**Nor**	2.72	0.52	644.01	0.00
**Owt**	4.47	0.29		
**Obe**	4.55	0.33		
**EOE**				
**Nor**	2.29	0.52	349.10	0.00
**Owt**	3.50	0.43		
**Obe**	3.76	0.38		
**DD**				
**Nor**	2.44	0.50	638.83	0.00
**Owt**	4.05	0.34		
**Obe**	4.32	0.41		
**‘Food Avoidance’ subscales**				
**SR**				
**Nor**	3.00	0.44	49.11	0.00
**Owt**	2.80	0.43		
**Obe**	2.35	0.69		
**SE**				
**Nor**	2.38	0.63	176.99	0.00
**Owt**	3.31	0.47		
**Obe**	3.79	0.63		
**EUE**				
**Nor**	3.04	334	98.28	0.00
**Owt**	2.53	65		
**Obe**	2.55	64		
**FF**				
**Nor**	3.07	334	36.03	0.00
**Owt**	2.67	65		
**Obe**	2.81	64		

Table 6 showed the comparison of mean score for the ‘Food Approach’ and ‘Food Avoidance’ subscales between the three groups of nutritional statuses of the children (normal weight, overweight and obese). The ‘Food Approach’ and ‘Food Avoidance’ subscales were significant in association with the three nutritional statuses. BMI: Body Mass Index; CEBQ: Children Eating Behaviour Questionnaires; DD: Desire to Drink; EF: Enjoyment of Food; EOE: Emotional Overeating; EUE: Emotional Undereating; FF: Food Fussiness; FR: Food Responsiveness; Nor: Normal; Obe: Obese; Owt: Overweight; SD: Standard Deviation; SE: Slowness in Eating; SR: Satiety Responsiveness.

## 4 Discussions

In view of rapid sociodemographic development in urban area, sedentary lifestyle and high availability of fast-food restaurants, we anticipated a high prevalence of obesity among primary school students around Kota Kinabalu city. However, the prevalence of obesity was only 13.2% which is lower compared to the prevalence in a more developed city in Klang city, which was 37% [10]. This finding was consistent with study conducted among 7 and 8 years old primary school students in 2012 in which the prevalence was 2.5% [11]. There was 13.4% overweight group which were not yet considered as obesity. This group of overweight primary school students are potential to become obese in future.

In this study, we found that the children's eating behaviour was strongly associated with their nutritional status. Children with excess weight had higher scores in all subscales that reflect “food approach” from the children eating behaviours questionnaires (CEBQ), in contrast with the children with a normal weight, where the “food approach” and “food avoidant” subscale were more evenly distributed. This finding supports our hypothesis where children with excess weight showed greater response to food, pleasure in eating, increased food intake due to the emotional state and a greater desire for beverages. In contrast to children with normal weight which showed weaker response to satiety and a pattern of faster food intake [4]. There were similar findings reported from previous studies where obese children exhibited stronger appetitive responses to food [4,5,12,13]. Apart from that, this study found that emotional overeating was positively corelated to the children BMI, which is similar to a study conducted by Jalo E et al. (2019) [14] who reported a positive association between emotional overeating and an unhealthy diet pattern. Foods consumed in response to negative emotions are usually high in sugar or fat and these palatable foods provide pleasure and instant reward, which may distract from the experience of negative emotion. On the other hand, scores of “food-avoidant” subscales which are the “Satiety responsiveness” and “emotional undereating and food fussiness” were inversely correlated with the children's body weight. In the SE ‘Food Avoidance’ subscale, the mean scores of overweight and obese groups were higher as compared to normal weight group. However, previous studies found that overweight children had lower scores on the subscale of Slowness in Eating (SE) which reflects a faster eating habit. Overweight children eat faster and with greater bite size compared with normal weight children [15]. This is inconsistent with our results. In term of age groups, the older the children, the more equally distributed the “food approach” and “food avoidant” mean score. Both genders showed a very similar eating behaviour with a difference in mean point only for the subscale of “enjoyment of food”, where the boys had higher score. This supports our finding where the boys (56.3%) had higher percentage of obesity compared to the girls (43.7%), which due to higher responsiveness to external food cues among the boys. Similar trend was reported by Passos DR et al. (2014) [4], where the only difference is in the mean score of desire to drink, and by Sirirassamee T et al. (2016) [16], in a cross-sectional study conducted in Thailand which demonstrated that boys scored higher on ‘enjoyment of food’ compared with girls as well as on ‘desire to drink’.

Our study has a several limitations. Firstly, it is difficult to determine the temporal associations between the factors as the study was cross-sectional in design. Secondly, the CEBQ is a parent-report measure and could be subject to bias. Finally, due to convenience sampling methods used in the school level to increase number of participants, the result produce may be biased in this study.

## 5 Conclusion

Although the prevalence of childhood obesity is low in the developing urban areas, it has a strong potential to be a significant healthcare burden in the future. This is because the children's eating behaviour is associated with their nutritional status, as evidence by the higher mean scores of ‘Food Approach’ subscales among the children in the overweight and obese groups as compared to the children in the normal weight group.

## Ethical approval

Ethical clearance has been obtained from the Universiti Malaysia Sabah Ethical Committee. The ethical approval code is JKEtika 2/18 (6).

## Sources of funding

This study is funded by 10.13039/501100006242Universiti Malaysia Sabah Research Grant Scheme (SGPUMS Fasa 1/2018, Grant code SLB0168-2018). The funding is use mainly for data collection, analysis and interpretation, and publication.

## Author contribution

Lily Shuzeen Kimin – drafting the article, gathering the team of authorship.

Constance Liew Sat Lin – supervisor and final approval of the version.

Richard Avoi – conception and design, or analysis and interpretation of data.

Firdaus Hayati - interpretation of data.

Mohd Nazri Mohd Daud - data collection and interpretation of data.

Alvin Oliver Payus –revising the article critically for important intellectual content, and final editing.

## Registration of research studies

Name of the registry:

Unique Identifying number or registration ID:

Hyperlink to your specific registration (must be publicly accessible and will be checked):

## Guarantor

Dr Alvin Oliver Payus.

## Consent

Written informed consent was obtained from all the subjects and their guardian for participation and publication of the data and accompanying images. A copy of the written consent is available for review by the Editor-in-Chief of this journal on request.

## Provenance and peer review

Not commissioned, externally peer reviewed.

## Declaration of competing interest

None.
